# Evaluation of the interfractional biological effective dose (BED) variation in MammoSite high dose rate brachytherapy

**DOI:** 10.1120/jacmp.v11i3.3228

**Published:** 2010-06-10

**Authors:** Yongbok Kim, E. Day Werts, Mark G. Trombetta, Moyed Miften

**Affiliations:** ^1^ Department of Radiation Oncology Allegheny General Hospital Pittsburgh; ^2^ Drexel University College of Medicine, Allegheny Campus Pittsburgh PA; ^3^ Department of Radiation Oncology University of Colorado Denver Aurora CO

**Keywords:** biological effective dose (BED), interfractional BED variation, MammoSite, HDR brachytherapy

## Abstract

The objective of this work is to evaluate the interfractional biological effective dose (BED) variation in MammoSite high dose rate (HDR) brachytherapy. Dose distributions of 19 patients who received 34 Gy in 10 fractions were evaluated. A method was employed to account for nonuniform dose distribution in the BED calculation. Furthermore, a range of α/β values was utilized for specific clinical end points: fibrosis, telangiectasia, erythema, desquamation and breast carcinoma. Two scenarios were simulated to calculate the BED value using: i) the same dose distribution of fraction 1 over fractions 2–10 (constant case, CC), and ii) the actual delivered dose distribution for each fraction 1–10 (interfraction dose variation case, IVC). Although the average BED difference (IVC – CC) was <0.7 Gy for all clinical endpoints, the range of difference for fibrosis and telangiectasia reached −11% to +3% and −9% to +9% for one of the patients, respectively. By disregarding high inhomogeneity in HDR brachytherapy, the conventional BED calculation tends to overestimate the BED for fibrosis by 16% on average, while it underestimates the BED for erythema (7.6%) and desquamation (10.2%). In conclusion, the BED calculation accounting for the nonuniform dose distribution provides a more clinically relevant description of the clinical delivered dose. Though the average BED difference was clinically insignificant, the maximum difference of BED for late effects can differ by a single fractional dose (10%) for a specific patient due to the interfraction dose variation in MammoSite treatment.

PACS number: 87.53.Jw

## I. INTRODUCTION

MammoSite high dose rate (HDR) brachytherapy has been widely used for early stage breast cancer patients.^(^ ^1^ ^)^ A spherically shaped dose distribution around the MammoSite balloon applicator (Hologic Corporation, Marlborough, Massachusetts, USA) can be easily reproduced. An intended dose can be delivered with high accuracy by a remotely controlled afterloader using a single plan for multifractional delivery.

In a previous multifractional HDR brachytherapy study,^(^ ^2^ ^)^ the deformity of the MammoSite balloon applicator from a sphere and the movement of a balloon toward the ipsilateral lung or skin during the course of HDR brachytherapy were measured. Additionally, the interfraction physical dose variations resulting from interfraction changes (shape and location) of the balloon applicator and trapped air gap volume were evaluated. In general, the interfraction physical dose variations were found to be clinically insignificant over the course of treatment. However, for certain fractions in some patients, clinically unacceptable dose variations may occur, such as less than 90% of target volume coverage and high doses to skin and ipsilateral lung. Adjustment of the MammoSite balloon applicator and replanning were suggested for those cases.

Measuring the interfraction dose variation with a biological metric can provide a better understanding of the overall biologic effect for the average interfraction dose variation as well as the potential high dose variation for certain treatment fractions in MammoSite delivery. In this work, the concept of biological effective dose (BED) was used to evaluate the interfraction dose variations resulting from the interfraction changes of the balloon applicator for 188 treatment fractions of 19 MammoSite HDR patients (patient 1 to 19). No computed tomography (CT) image data were available for two treatment fractions. This study was approved by the institutional review board.

## II. MATERIALS AND METHODS

### A. BED calculation

The cell survival (S) fraction from the total dose of “D” delivered in the number of fractions of “n” with fractional dose of “d” is known as following equation based on the linear‐quadratic (LQ) model:^(^ ^3^ ^,^ ^4^ ^)^

(1) $$S=\text{exp}[-\alpha \text{nd}-\beta {\text{nd}}^{2}]=\text{exp}\left[-\alpha \text{nd}\left(1+\frac{d}{\alpha /\beta }\right)\right]$$

The “αnd” and “$\beta {\text{nd}}^{2}$” are the linear and quadratic components of cell killing and α (in units of ${\text{Gy}}^{-1}$) and β (in units of ${\text{Gy}}^{-2}$) are the radiosensitivity coefficients for each component. If the cell repopulation is taken into account as the second term, the Eq. (1) becomes the following equation:

(2) $$S=\text{exp}\left[-\alpha \text{nd}\left(1+\frac{d}{\alpha /\beta }\right)+\frac{(\ln\;2)T}{T_{\text{eff}}}\right]$$

$T_{\text{eff}}$ is the effective cell doubling time over the treatment time T. Allowance for a delay time ($T_{\text{delay}}$) is not included in the equation, so the doubling time to be used is the average over the given treatment duration. Note that the second term is not needed for late‐responding tissue since cell repopulation does not usually occur in these tissues during the course of irradiation. Traditionally, the BED method has been used to assess the biological effectiveness (E) of a dose “D” to the irradiated cells by the relationship of BED=E/α=(ln S)/α. Therefore, the conventional BED formulism (${\text{BED}}_{C}$: Conventional method) can be written in Eq. (3):

(3) $${\text{BED}}_{C}=\text{nd}\left(1+\frac{d}{\alpha /\beta }\right)-\frac{(\text{ln}\;2)T}{\alpha T_{\text{eff}}}$$

The biological effectiveness for a certain fractionation scheme is represented by the value of “1+d/(α/β)”, depending upon the fractional dose “d” and “α/β” (the dose at which the linear and quadratic components of radiation damage are equal). The α/β ratio depends on the characteristics of the tissue of interest and is measured in units of Gy.

**Figure 1:**
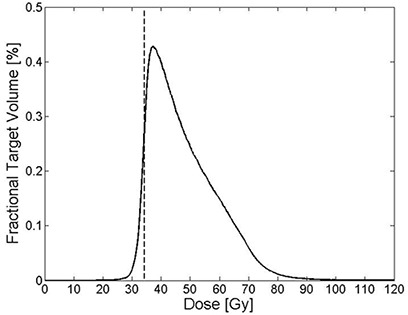
Group average differential dose volume histogram (dDVH) for 19 MammoSite patients. The vertical line represents the prescribed dose of 34 Gy and the size of dose bin is 0.1 Gy. The group average dDVH is computed by averaging fractional target volume corresponding to each dose bin for 19 patients.

The underlying hypothesis of the conventional BED calculation (${\text{BED}}_{C}$) is that all cells of interest receive the same dose as the prescribed dose throughout the treatment and the cell repopulation term is only affected by the treatment time T for a specific tissue. However, the prescribed dose cannot be shaped ideally to cover the entire target volume in a clinical treatment plan and all target cells cannot receive exactly the same dose as the prescribed dose. Moreover, the delivered dose distribution for a specific treatment fraction cannot be the same as the planned dose distribution. For example, the group average^(^ ^5^ ^,^ ^6^ ^)^ differential dose volume histogram (dDVH) calculated using a dose bin size of 0.1 Gy for the 19 MammoSite HDR patients (Fig. 1) shows that the prescribed dose of 34 Gy (33.9 Gy<Dose≤34.1 Gy in Table 1) was only delivered to 0.49% of the target volume while the majority of target volume received much higher dose. The most probable (modal) and mean dose to the target were 37.7 Gy and 48.0 Gy for the 19 patients, on average. In dDVH, the sum of the fractional target volume for all dose ranges is 100% and the fractional target volume receiving a certain dose varies depending upon the size of dose bin.

**Table 1:** Part of group average differential dose volume histogram (dDVH) table for 19 MammoSite patients (Fig. 1) with dose bin size 0.1 Gy.

*Dose Range* a	*Fractional Target Volume (%)*
33.8 Gy < Dose ≤33.9 Gy	0.229
33.9 Gy < Dose ≤34.0 Gy	0.239
34.0 Gy < Dose ≤34.1 Gy	0.251
34.1 Gy < Dose ≤34.2 Gy	0.264

a The dose range from 33.8 to 34.2 Gy was selectively chosen in the vicinity of the prescribed dose of 34 Gy to demonstrate that only 0.49% of target volume received the prescribed dose (33.9 Gy < Dose ≤34.1 Gy).

In order to account for the nonuniform dose distribution (heterogeneity) within the target volume, we utilized a more clinically relevant approach for the BED calculation (${\text{BED}}_{H}$: Heterogeneity method). Specifically, the BED value was calculated for a certain subvolume within the target volume where dose distribution was uniform although it was heterogeneous for the entire target volume. The BED value for the specific subvolume of “i” can be computed using the cell survival fraction of subvolume i by ${\text{BED}}_{C,i}=-(\ln\;s_{i})/\alpha$. Hence, the overall ${\text{BED}}_{H}$ value is computed by summing the volume weighted survival fraction over the entire target volume as follows in Eq. (4):^(^ ^7^ ^–^ ^9^ ^)^

(4) $${\text{BED}}_{H}=-\frac{1}{\alpha }\text{ln}\left(\sum_{i=1}^{N}v_{i}s_{i}\right)$$

where $v_{i}$ is fraction of the subvolume in the target and the sum of all fractional subvolumes is unity. N is the total number of dose bins. However, it is difficult to specify the subvolume within the target whose dose distribution is uniform. Instead, the dDVH was employed in this study. All fixed size dose bins are selected first and their corresponding subvolume(s) are determined accordingly. If a specific nonuniform dose distribution within the target volume remains unchanged (CC: constant case) throughout the full course of radiation delivery, the total ${\text{BED}}_{H,t}^{\text{CC}}$ value for the dose of “D” delivered in the total number of fraction “n” can be computed as follows.

(5) $${\text{BED}}_{H,t}^{\text{CC}}=-\frac{1}{\alpha }\text{ln}\left\{\sum_{i=1}^{N}v_{i}\text{exp}\left[-\alpha \;n\;d_{i}\left(1+\frac{d_{i}}{\alpha /\beta }\right)\right]\right\}-\frac{(\ln\;2)T}{\alpha T_{\text{eff}}}$$

where $d_{i}$ and $v_{i}$ are the dose in each bin and its fractional volume. However, in a clinical practice, the nonuniform dose distribution varies according to the change of patient anatomy as well as applicator geometry for each fraction. Consequently, a fractional ${\text{BED}}_{H,f}^{\text{IVC}}$ value for a certain fraction “f” which has a specific nonuniform dose distribution can be computed by the following equation:

(6) $${\text{BED}}_{H,f}^{\text{IVC}}=-\frac{1}{\alpha }\text{ln}\left\{\sum_{i=1}^{N}v_{i}\text{exp}\left[-\alpha \;d_{i}\left(1+\frac{d_{i}}{\alpha /\beta }\right)\right]\right\}-\frac{(\ln\;2)T}{n\;\alpha T_{\text{eff}}}$$

Because the cell repopulation term is not related to the dose distribution for each fraction, the effect of cell repopulation term was considered to be constant for each fraction. More clinically relevant ${\text{BED}}_{H,t}^{\text{IVC}}$ value for the dose of “D” delivered in “n” treatment fractions can be calculated by the summation of the fractional ${\text{BED}}_{H,f}^{\text{IVC}}$ value for each fraction with its specific nonuniform dose distribution (IVC: interfraction variation case).

(7) $${\text{BED}}_{H,t}^{\text{IVC}}=\sum_{f=1}^{n}{\text{BED}}_{H,f}^{\text{IVC}}$$

If a nonuniform dose distribution is the same throughout the full course of treatment, the ${\text{BED}}_{H,t}^{\text{CC}}$ value from Eq. (7) will be exactly the same as the value from Eq. (5).

### B. Treatment planning for fraction 1

**Figure 2:**
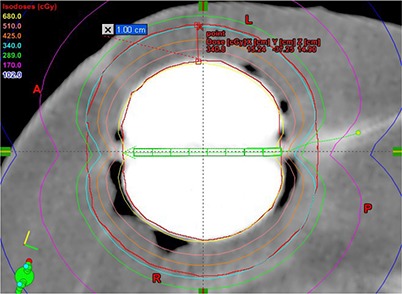
Two‐dimensional CT image to show a typical MammoSite balloon with isodose distribution for patient 19. The volume of PTV was the same with PTV_EVAL for this patient because the balloon was located in the middle of breast and the volume of skin +5 mm and lung/pectoralis muscle did not interfered with the volume of balloon +1 cm. Eight possible dwell positions were used along the straight MammoSite balloon catheter.

A commercial treatment planning system (TPS) (BrachyVision V6.5, Varian Medical Systems Inc, Charlottesville, Virginia, USA) was used for computed tomography (CT) image based HDR planning. On the axial CT images acquired prior to fraction 1, the planning target volume (PTV) and PTV for plan evaluation purpose (PTV_EVAL) were defined according to the National Surgical Adjuvant Breast and Bowel Project (NSABP) B‐39/ Radiation Therapy Oncology Group (RTOG) 0413 protocol.^(^ ^10^ ^)^ The PTV was defined as a spherical shell with 1 cm thickness. Balloon surface was expanded with 1 cm in three‐dimensions (3D) and the PTV was obtained by extracting the balloon volume from the 1 cm expansion. The average diameter of the balloon for 19 patients was 4.6 cm ranging from 4.0 cm to 5.5 cm. Therefore, the number of available dwell positions was different for each patient depending upon the diameter of the balloon and ranged from 7 to 10. The PTV_EVAL volume was constructed the same as the PTV except for excluding the volumes of skin +5 mm and lung/pectoralis muscle. To reduce the uncertainty in localizing the catheter lumen inside the MammoSite balloon, the CT images were rotated in 3D, and their contrast and brightness were modified to best visualize the lumen inside the balloon. The desired fractional dose of 3.4 Gy was prescribed to dose grid points located on the surface of the 1 cm expansion of the balloon in 3D. To minimize the high dependency of dose distribution on the location of a single dwell position at the center of balloon, a multiple dwell position approach was used for all patients. Figure 2 shows the dwell positions for patient 19. In this example, eight dwell positions were available to optimize the dwell time distribution. In addition, the surface optimization technique in the commercial TPS was utilized for optimizing the dwell time distribution to achieve better target dose coverage.^(^ ^11^ ^–^ ^13^ ^)^ The optimal dwell time distribution was different depending upon the geometry of the balloon for each patient. The resultant dose distribution was shaped to cover 1 cm expansion from the balloon (as shown in Fig. 2). There was a cold spot along the axis of the balloon catheter due to the characteristic of line source.

### C. BED calculation for interfraction dose variation

Clinically, the treatment plan of fraction 1 is used for the treatment of fractions 2–10 without any modification unless a significant change of the MammoSite applicator is observed such as a rupture of the balloon, a large change (i.e. >10%) in the air gap volume relative to the PTV_EVAL, or noticeable movement of the balloon between fractions when comparing the prefractional treatment CT images with the planning CT images. To mimic this clinical practice, we assumed in this work that the shape and location of MammoSite balloon applicator are reproducible for each fraction 1–10 and the interfraction dose variation can be ignored. The same nonuniform dose distribution of fraction 1 is employed for all fractions 1–10, and in this constant case (CC) the ${\text{BED}}_{t}^{\text{CC}}$ value is computed using Eq. (5).

To account for the interfraction dose variation, the actual delivered dose distribution for each fraction should be employed in the fractional ${\text{BED}}_{H,f}^{\text{IVC}}$ calculation (Eq. (6)). To simulate this interfraction variation case (IVC), a plan was retrospectively generated for each fraction 2–10. The PTV_EVAL and critical structures were delineated and the balloon catheter was also identified on the CT images taken prior to each fraction 2–10. The dwell time distribution of the fraction 1 plan was manually transferred to the plans for fractions 2–10 to mimic the clinical situation where the fraction 1 plan is employed for fraction 2–10 without modification. To eliminate errors during the manual transfer process, the plan report of fractions 2–10 was validated in comparison with that of fraction 1. Consequently, the dose distribution of fraction 1 was implemented on the target and critical structures of fractions 2–10. If there is no interfraction variation of the MammoSite balloon applicator, the dose distribution of the target and critical structures would be the same for fractions 1–10. However, in reality, as the shape or location of MammoSite balloon applicator changes, the dose received by the target and critical structures varies and is different for each fraction. The change in delivered dose to the target and critical structures depends on the magnitude of the change in the shape and/or location of the MammoSite applicator. A total of 188 plans (19 plans for fraction 1 and 169 plans for fraction 2–10) were generated for 19 patients. A CT scan was unavailable for fraction 10 of patient 4 and fraction 6 of patient 6. The ${\text{BED}}_{t}^{\text{IVC}}$ value in this IVC was computed by the summation of the different fractional ${\text{BED}}_{H,f}^{\text{IVC}}$ values over fractions 1–10 (Eq. (7)) using the different dose distribution for each fraction.

### D. Clinical endpoints and biological parameters for BED calculation

Among various normal tissue endpoints, two acute (erythema and desquamation) and two late (fibrosis and telangiectasia) effects were evaluated. Those acute and late effects were clinically the most common and widely investigated in many toxicity and cosmesis outcome studies^(^ ^14^ ^–^ ^21^ ^)^ following MammoSite treatments. The BED values for breast carcinoma were also calculated. As seen in Eq. (3), the BED value was calculated using the specific radiobiological parameters such as α/β ratio, α value and value of $T_{\text{eff}}$. The α/β ratio was adopted from the literature for specific clinical endpoints.^(^ ^21^ ^–^ ^25^ ^)^ For acute normal tissue effects, α/β values of 8 Gy and 11 Gy were used for erythema and desquamation, respectively. For late normal tissue effects, α/β values of 2 Gy and 4 Gy were used for fibrosis and telangiectasia, respectively. An α/β value of 4 was used for breast carcinoma.^(^ ^21^ ^,^ ^26^ ^)^ An α value of 0.3 ${\text{Gy}}^{-1}$ and $T_{\text{eff}}$ value of 13 days were used for the cell repopulation term.^(^ ^14^ ^,^ ^27^ ^)^

### E. Comparison of BED calculation methods

As shown in Fig. 1, MammoSite plans have a marked nonuniform dose distribution in the target volume which may potentially vary throughout the treatment. While the heterogeneity is accounted for in the BED calculation in this work using either ${\text{BED}}_{H,t}^{\text{CC}}$ or ${\text{BED}}_{H,t}^{\text{IVC}}$ (H‐method), it is disregarded in the conventional BED calculation (C‐method). Therefore, assessing the discrepancy of BED values between H‐method and C‐method can demonstrate the characteristics of both BED calculation methods for various α/β ratios. The BED value (${\text{BED}}_{H,t}^{\text{CC}}$) using H‐method (Eq. (5)) was compared with ${\text{BED}}_{C,t}$ value using C‐method (Eq. (3)) for 19 MammoSite patients. Equation (3) assumes that the entire target volume received the prescribed dose, while Eq. (5) accounts for the heterogeneity of target dose distribution for each patient. For a consistent comparison between H‐method and C‐method, the heterogeneity of target dose distribution was assumed to be unchanged over the fractions in the H‐method.

## III. RESULTS

**Figure 3:**
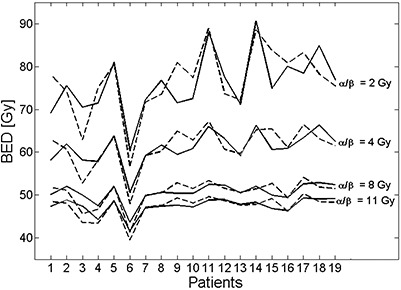
Comparison of BED values over various α/β ratios between CC (solid lines) and IVC (dash lines) for 19 MammoSite patients. The BED value was computed over 9 fractions for patients 4 and 6, and over 10 fractions for the remaining 17 patients.

Figure 3 shows the BED values of CC (from Eq. (5)) and IVC (from Eq. (7)) cases for 19 MammoSite patients over the range of α/β ratios from 2 Gy to 11 Gy. The average ± standard deviation (SD) value of the difference (IVC – CC) in BED values is summarized with minimum and maximum values in Table 2. Negative value means the BED value for IVC is smaller than CC. Although the average difference was clinically insignificant (<0.7 Gy), the deviation was so patient‐specific and large in particular for late effects that it could not be disregarded. In general, with smaller α/β ratio, larger deviation was observed. The SD value of BED difference was 6.9% for fibrosis (α/β ratio of 2 Gy) and 4.8% for telangiectasia and breast carcinoma (α/β ratio of 4 Gy) relative to CC. Consistently, the BED difference (IVC – CC) was most negative in patient 3 for all clinical endpoints (−10.7%, −9.3%, −8.7% and −8.1% for α/β ratios of 2, 4, 8 and 11 Gy, respectively, relative to CC). Patient 9 showed the most positive value of BED difference for α/β ratios of 2 Gy (13.1%) and 4 Gy (9.2%), while patient 15 for α/β ratios of 8 Gy (5.7%) and 11 Gy (5.0%).

**Table 2:** The data of 19 patients showing the difference (IVC ‐ CC) in BED values between the interfraction variation case (IVC) and the constant case (CC) for specific clinical endpoints.

*Clinical Endpoint*	α*/*β *(Gy)*	*Average (Gy)*	*Standard deviation*	*Minimum (Gy)*	*Maximum (Gy)*
Fibrosis	2	0.6	5.0	−7.6 (3)^a^	9.4 (9)
Telangiectasia	4	0.2	2.9	−5.4 (3)	5.5 (9)
Erythema	8	−0.1	1.6	−4.3 (3)	2.8 (15)
Desquamation	11	−0.1	1.4	−3.9 (3)	2.4 (15)
Breast carcinoma	4	0.2‐	2.9	−5.4 (3)	5.5 (9)

a ( ) identifies specific patient number

**Figure 4:**
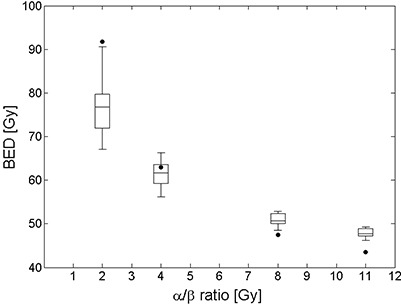
Comparison of BED values computed by uniform dose distribution using the C‐method (Eq. (3)) (black circle) and nonuniform dose distribution using the H‐method (Eq. (5)) (box graph) for 19 MammoSite patients. Because the BED value is different for each patient in the H‐method, the calculated BED values are shown as a box graph in which each parallel bar represents minimum, 25, 50, 75 percentile and maximum value for 19 patients.

Figure 4 shows the BED value comparison between the H‐method (Eq. (5)) and C‐method (Eq. (3)). H‐method regularly resulted in lower BED values for fibrosis (α/β ratio of 2 Gy) and higher BED values for early effects such as erythema (α/β ratio of 8 Gy) and desquamation (α/β ratio of 11 Gy) compared to the C‐method. For the α/β ratio of 4 Gy (telangiectasia and breast carcinoma), the BED value by the H‐method was comparable to those by the C‐method and patient‐specific depending on the heterogeneous dose distribution of the patient. The comparison for 19 MammoSite patients is summarized in Table 3 with some statistics (mean, standard deviation, minimum and maximum values). Furthermore, the difference of BED value $\left({\text{BED}}_{H}-{\text{BED}}_{C}\right)$ as well as its relative difference $\left({\text{BED}}_{H}-{\text{BED}}_{C})\times 100/{\text{BED}}_{C}[\%\right]$ was analyzed and summarized in Table 4. On average, the H‐method produced lower BED values for fibrosis (α/β ratio of 2 Gy) by 16.4% and higher BED values for early effects such as erythema (α/β ratio of 8 Gy) by 7.6% and desquamation (α/β ratio of 11 Gy) by 10.2%, respectively, compared to the C‐method. For the α/β ratio of 4 Gy (telangiectasia and breast carcinoma), the average relative difference of BED value was −2% (range from −10.6% to 5.5%).

**Table 3:** Comparison of BED value computed using uniform dose distribution (C‐method of Eq. (3)) and nonuniform dose distribution (H‐method of Eq. (5)) for 19 MammoSite patients.

*Clinical Endpoint*	α/β *(Gy)*	*BED Value (Gy) using C‐method* a	*BED Value (Gy) using H‐method* b			
			*Average*	*Standard Deviation*	*Minimum*	*Maximum*
Fibrosis	2	91.8	76.7	6.3	67.0	90.6
Telangiectasia	4	62.9	61.7	2.9	56.2	66.3
Erythema	8	47.5	51.1	1.3	48.5	52.9
Desquamation	11	43.5	48.0	1.0	46.2	49.3
Breast carcinoma	4	62.9	61.7	2.9	56.2	66.3

a The C‐method used the prescribed dose as the uniform dose distribution for all fractions and thus the BED value was the same for all patients.

b The H‐method used the delivered nonuniform dose distribution of fraction 1 for all fractions and the BED value was different for each patient depending upon its nonuniform dose distribution. Hence, the average, minimum, and maximum along with the standard deviation value are reported for BED value using H‐method.

**Table 4:** BED value difference $\left({\text{BED}}_{H}-{\text{BED}}_{C}\right)$ and the relative BED difference normalized to the ${\text{BED}}_{C}$ for 19 MammoSite patients.

*Clinical Endpoint*	α/β *(Gy)*	*Average*	*Standard Deviation*	*Minimum*	*Maximum*
BED Difference [Gy]: ${\text{BED}}_{H}$ – ${\text{BED}}_{C}$					
Fibrosis	2	−15.1	6.3	−24.8	−1.2
Telangiectasia	4	−1.2	2.9	−6.7	3.4
Erythema	8	3.6	1.3	1.0	5.4
Desquamation	11	4.5	1.0	2.7	5.8
Breast‐Carcinoma	4	−1.2	2.9	−6.7	3.4
Relative BED Difference $[\%]:(\frac{{\text{BED}}_{H}-{\text{BED}}_{C}}{{\text{BED}}_{C}})\times 100$					
Fibrosis	2	−16.4	6.9	−27.0	−1.3
Telangiectasia	4	−2.0	4.6	−10.6	5.5
Erythema	8	7.6	2.8	2.1	11.3
Desquamation	11	10.2	2.3	6.2	13.3
Breast Carcinoma	4	−2.0	4.6	−10.6	5.5

## IV. DISCUSSION

A specific clinical endpoint results from the radiation dose to a specific tissue. For instance, consider when the same radiation dose is delivered to two different types of tissues. One tissue develops a certain clinical endpoint while the other tissue responds with a different clinical endpoint depending upon the characteristics of each tissue. Skin tissue of the breast may respond with either erythema or desquamation. Some connective tissues in the breast form a development of fibrosis and some blood vessels can develop telangiectasia after irradiation. Also, the radiation dose kills breast cancer cells. In the clinical planning, the volume of PTV_EVAL may consist of the mixture of many types of tissues for a particular patient, as reported by Rosenstein et al.^(^ ^21^ ^)^ In an ideal situation where each type of tissue can be exclusively identified in the breast and delineated on the planning CT images, tissue‐specific DVH can be generated for each volume of tissue type. However, with current MammoSite planning systems it is impossible to extract those tissue‐specific DVH data. In this study, the volume of PTV_EVAL was considered as a unique volume containing all type of tissues corresponding to all clinical endpoints. Consequently, the same dDVH for PTV_EVAL was used in the BED calculation for all type of clinical endpoints.

Even though the average BED difference stemming from interfraction dose variation was <0.7 Gy for 19 patients, some patient‐specific deviation was significant. The maximum deviation was from −11% to +13% for late responding effects (α/β ratio of 2 Gy) and from −9% to +6% for early responding effects (α/β ratio of 8 or 11 Gy). Therefore, interfractional physical dose variation for a specific patient can make the total BED differ by 10% for a certain clinical endpoint which corresponds to the single fractional dose out of 10 total fractions.

By disregarding high inhomogeneity in HDR brachytherapy, the conventional simple calculation tends to overestimate the BED value for fibrosis while underestimating the BED value for erythema and desquamation. Hence, in order to evaluate the clinical endpoints, our data suggest it is more clinically relevant to utilize the BED calculation accounting for the dose heterogeneity in target volume. However, one must stress that the BED and the underlying LQ model were derived from cell survival curves generated from uniform irradiation, and therefore we must be cautious when using the BED concept.

The high heterogeneity of target dose can be anticipated in MammoSite plans because an Ir‐192 source mostly located at the center of the balloon yields a spherically shaped dose distribution with a gradual dose fall‐off.^(^ ^28^ ^)^ In general, the average dose is more than 200% of the prescribed dose at the surface of the balloon and it is gradually reduced to the prescribed dose at the 1 cm expansion of balloon surface. Hence, the maximum dose in the target is always more than 200% of the prescribed dose for MammoSite planning. As the α/β ratio is increased from 2 Gy to 11 Gy, the standard deviation of BED value (from the H‐method) between patients was reduced from 7% to 2% (Table 4). This can be interpreted to mean that target dose heterogeneity in MammoSite plans is less patient‐dependent for early effects than late effects.

Although the inhomogeneity of the target volume was accounted for in the BED calculation, one cannot explain the radiation dose response and predict the clinical outcomes exactly based on the computed BED value. This simplified mathematical model cannot describe all tissue responses to a given radiation dose. In addition, the biologic parameters such as α/β ratio, αvalue and value of $T_{\text{eff}}$ used in the BED calculation have intrinsic uncertainty even though the values used in this work were based on clinical data in the literature. Despite these limitations, the BED model is the best available tool we have at present. An extensive clinical follow‐up study, such as the NSABP B‐39 / RTOG 0413 protocol, will afford a greater opportunity to assess the computed BED values and correlate these with clinical outcomes. However, the computed BED values from the protocol will be based on the uniform dose distribution because it is very difficult to account for the heterogeneous dose distribution for the multi‐institutional protocol patients.

## V. CONCLUSIONS

The BED calculation accounting for nonuniform dose distribution is more clinically relevant compared to the conventional simple method, assuming uniform dose distribution across the target volume. By disregarding high inhomogeneity in HDR brachytherapy, the conventional calculation tends to overestimate the BED for fibrosis while it underestimates the BED for erythema and desquamation. Based on the BED analysis for given α/β values, the interfraction physical dose variations due to the changes of balloon shape and location in MammoSite HDR brachytherapy can produce BED difference by 10% (a single fraction out of total 10 fractions) for a specific patient even though the average difference for 19 patients was less than 0.7 Gy.
